# STIGMA IN THE CONTEXT OF ALZHEIMER’S DISEASE AND RELATED DEMENTIAS (ADRD) WITHIN RURAL AND UNDERSERVED POPULATIONS

**DOI:** 10.1093/geroni/igac059.187

**Published:** 2022-12-20

**Authors:** Elizabeth Rhodus, Steffi Kim, Fayron Epps

## Abstract

Stigma in the context of Alzheimer’s disease and related dementias (ADRD) is associated with a higher prevalence of depression, anxiety, social isolation, and poorer caregiver health. This is particularly true for underserved and rural communities; however, little is known about the sources of stigma and implications of stigma within these communities. This symposium explores sources of stigma along with implications of stigma in rural and/or underserved communities and introduces novel interventional considerations for addressing stigma. The first presentation by Rhodus and colleagues highlights implications of stigma in rural Appalachian communities as it relates to ADRD healthcare service and research participation. Next, Sabat and colleagues present findings of a recent intervention program, “Respite for All,” specifically, implications of this program for caregivers’ perception of stigma, as well as the person living with ADRD. This symposium also includes presentations focused on Alaska Native (AN) experiences with stigma and ADRD. Kim discusses findings of a community-based participatory research project using mixed-method to explore structural stigma in rural communities and needed initiatives for familial care partners. To conclude the program, Crouch and Rosich present results of a grounded theory, exploratory study aimed to understand the cultural practices and values that compose AN Elder beliefs and perceptions of ADRD, including stigmas. This symposium will conclude with a discussion on how researchers may begin to integrate approaches to address stigma in rural and underserved communities in order to enhance care utilization and quality of life for older adults caring for and living with ADRD.

